# Visuotactile object processing in binocular rivalry: The role of shape congruence, voluntary action, and spatial colocalization

**DOI:** 10.1167/jov.25.14.11

**Published:** 2025-12-17

**Authors:** Seyoon Song, Haeji Shin, Chai-Youn Kim

**Affiliations:** 1 School of Psychology, Korea University, Seoul, Republic of Korea

**Keywords:** binocular rivalry, visuotactile integration, three-dimensional shape, voluntary action, shape congruence, spatial colocalization

## Abstract

Multisensory information can help resolve perceptual ambiguity in situations such as the alternating visual experience during binocular rivalry. Across four experiments, participants viewed dichoptically presented spiky and round rival targets while simultaneously touching spiky, neutral, or round shapes in three-dimensional (3D) printed form. The primary aim was to investigate the influence of visuotactile shape congruence in the curvature dimension. In addition, the roles of voluntary action and spatial colocalization on successful crossmodal integration were investigated. Voluntary action was tested between active touch (Experiments 1 and 2) and passive touch (Experiments 3 and 4) conditions. Visual stimulus type differed between rapid successions of 3D-rendered images (Experiments 1 and 3) and real-world video recordings (Experiments 2 and 4), with the latter involving bodily cues to promote visuotactile colocalization. In general, the results showed that tactile shape congruence can lead to relative dominance of the corresponding visual target, especially when visuotactile colocalization was encouraged with video recordings as visual targets. The results suggest beneficial effects of crossmodal shape congruence on disambiguation, which seems to be generally comparable between the two modes of active versus passive touch. Using 3D stimuli and including free voluntary action, the study provides novel and connecting insights into the naturalistic object processing behavior of humans.

## Introduction

The nature of the world surrounding us is multisensory. Multisensory integration refers to the process of combining senses to create unified perceptual experiences, and multimodal activation is considered more effective than its unimodal components (Alais, Newell, & Mamassian, 2010; Driver & Spence, 2000; Stein & Stanford, 2008). An important role of multisensory integration is perceptual disambiguation—the process that mitigates ambiguity from the environment to form a coherent percept of the world (Attneave, 1971; von Helmholtz, 1925). This characteristic of perception is well exemplified by bistable perception, in which two subjective interpretations can exist for a single physical input, thus inducing spontaneous alternations between the two percepts (Kim & Blake, 2005; Leopold & Logothetis, 1999). In such ambiguous situations of bistable or even multistable perception, when a signal from a single sensory modality is insufficient to create a robust percept, other sensory modalities can help make the percept clearer (Ernst & Bülthoff, 2004; Green & Angelaki, 2010; Lee, Blake, Kim, & Kim, 2015).

Among possible crossmodal combinations, the main topic of the current study is the interaction between vision and touch. Existing literature has generally suggested that, in visual multistability situations, concurrent stimulation in touch can aid in disambiguation toward the congruent visual percept (Blake, Sobel, & James, 2004; Bruno, Jacomuzzi, Bertamini, & Meyer, 2007; van Ee, Boxtel, Parker, & Alais, 2009). However, studies have mostly evaluated one-dimensional (1D) or two-dimensional (2D) stimuli, such as sinusoidal gratings or simple motions (Hense, Badde, & Röder, 2019; Liaw, Kim, & Alais, 2022; Lunghi & Alais, 2013; Lunghi & Alais, 2015; Lunghi, Binda, & Morrone, 2010; Lunghi & Morrone, 2013; Lunghi, Morrone, & Alais, 2014; van Ee et al., 2009). Vision and touch are both renowned for their superiority in three-dimensional (3D) object processing (Klatzky, Lederman, & Metzger, 1985; Lacey & Sathian, 2014; Lederman & Klatzky, 1987; Newell, Ernst, Tjan, & Bülthoff, 2001), which makes the common stimuli designs in the previous literature a significant shortcoming. In this context, the study of Blake et al. (2004) is noteworthy because it introduced a 3D tactile globe matching the visually bistable stimulus, which is closer to the properties of everyday objects. Still, more studies using 3D stimuli could lead to richer insights regarding the process of visuotactile integration.

Another important component in visuotactile object recognition is the voluntary action of the perceiver. In previous literature, static contacts or monotonous finger strokes were commonly instructed (Blake et al., 2004; Hense et al., 2019; Liaw et al., 2022; Lunghi & Alais, 2013; Lunghi & Alais, 2015; Lunghi et al., 2010; Lunghi et al., 2014; van Ee et al., 2009). However, in real-world situations, perceivers can lift a 3D object and hold it in their palms or enclose it tightly in their hands and feel its contour (Lederman & Klatzky, 1987). Indeed, haptic perception involving exploratory motor activity, which is henceforth referred to as active touch, can be dissociated from tactile perception with passive touch (Gibson, 1962; Reed & Ziat, 2018). Particularly regarding 3D stimuli, a variety of hand movements can be made; thus, the importance of investigating the influence of voluntary action is raised to better understand the mechanisms underlying object recognition.

To sum up, regarding naturalistic 3D object recognition, the effects of visuotactile congruence on visual disambiguation is yet to be studied. To this end, binocular rivalry was selected as the experimental tool to induce perceptual ambiguity. In binocular rivalry, two different images are presented dichoptically to each eye, and what is viewed as dominant alternates back and forth between the two eyes (Blake & Logothetis, 2002; Levelt, 1965). This phenomenon allows the systematic examination of processes governing perceptual competition, neural dynamics, and selection of the contents of visual awareness (Alais & Blake, 2005). In general, previous literature has reported longer dominance and shorter suppression toward visual targets with congruent touch, suggesting beneficial impacts of visuotactile integration in binocular rivalry (Lunghi & Alais, 2015; Lunghi & Morrone, 2013; Lunghi et al., 2010; van Ee et al., 2009).

The present study used 3D objects as experimental stimuli during binocular rivalry to understand the influence of visuotactile integration on perceptual disambiguation. Across four experiments, congruence in vision and touch was manipulated by shape curvature. Novel 3D stimuli of spiky, round, and neutral shapes were used. Spiky and round stimuli were dichoptically introduced in binocular rivalry, whereas tactile presentations of the spiky, neutral, or round stimulus in 3D-printed form were simultaneously provided. Shape congruence between the visual and tactile stimuli was the main concern in the current study. In addition, the role of voluntary action was investigated based on active touch (Experiments 1 and 2) and passive touch (Experiments 3 and 4). Moreover, visual stimulus types were varied between 3D-rendered images (Experiments 1 and 3) and video recordings including a touching hand (Experiments 2 and 4) to test the influence of spatial colocalization in visuotactile integration. The general hypothesis was that concurrent tactile stimulation will lead to dominance of congruent visual shape in binocular rivalry, along with the investigation of additional factors such as the presence of voluntary action or spatial colocalization.

## Experiment 1

The aim of the first experiment was to study the effects of visuotactile integration on binocular rivalry dynamics while participants actively explored tactile stimuli. While rapid successions of 3D-rendered images of spiky and round shapes were presented dichoptically through the binocular rivalry paradigm, a spiky, neutral, or round 3D-printed object was presented for the participants to freely explore in their palms with their fingers. The hypothesis was that active touch experience of a certain shape would bring the congruent visual percept into dominance during binocular rivalry.

### Methods

#### Participants

Sixteen individuals (six women; mean age, 28.1 ± 3.0 years; range, 24–33 years) with normal or corrected-to-normal vision participated in the experiment. Because of the design and aim of the experiment to explore the effect of visuotactile integration when touching 3D stimuli, only right-handed participants were recruited. Participants showing strong eye dominance were excluded from the analyses. The study was approved by the Korea University Institutional Review Board (KUIRB-2019-0313-02).

#### Apparatus

The experiment was conducted in a quiet, dark room. Participants sat in front of a 19-inch cathode ray tube (CRT) monitor (1024 × 768-pixel resolution, 60-Hz refresh rate, 52-cm viewing distance), and the experiment was conducted using MATLAB 2016 (MathWorks, Natick, MA) and Psychophysics Toolbox 3 (Brainard, 1997; Pelli, 1997).

#### Stimuli

**Figure 1.**
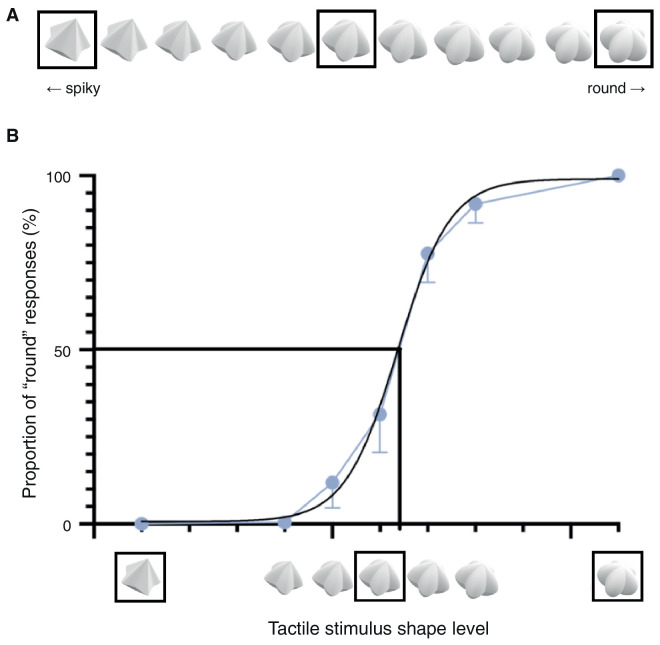
(**A**) Eleven shape stimuli ranging from the spikiest to the roundest were created using a parametric shape model. The three boxed stimuli were chosen to be used in the main experiments: spiky shape (left), neutral shape (middle), and round shape (right). (**B**) The results of the preliminary experiment conducted to choose and validate the stimuli. A psychometric curve is fitted on the proportion of “round” responses collected from the 2AFC task. Seven stimuli used in the preliminary experiment are shown on the *x*-axis. The three boxed stimuli were chosen to be used in the main experiments.

Novel 3D stimuli with various curvatures were created using a parametric shape model (“superformula”) (Gielis, 2003; Kwak, Nam, Kim, & Kim, 2020; Kwak, Nam, & Kim, 2018). Spiky and round shapes were created initially, with similar base shapes of five protruding arms. Using the coordinates of the shape models, 11 shapes varying gradually from spiky to round were created via linear interpolation (Figure 1A). A preliminary experiment was conducted to validate that these mathematically created stimuli were indeed perceived in the intended roundness (Figure 1B). Among the 11 shapes, seven shapes were used in the psychophysics experiment where participants explored the shape stimuli in their hand and performed a two-alternative, forced-choice (2AFC) task of determining whether the perceived shape was spiky or round. When fitted with a psychometric curve, the results indicated that the two extremes of the shape model were indeed perceived as the spikiest and the roundest and thus were chosen as the spiky and round stimuli for the experiment. Moreover, the shape closest to the equal proportion of spiky and round responses was chosen as the neutral stimulus (Figure 1).

As for the visual stimuli, spiky and round stimuli were 3D-rendered using MATLAB. Each stimulus was placed on a fixed axis and illuminated so it would sufficiently show the shape from a typical point of view. Snapshots were captured every 2° of rotation, resulting in a total of 180 rendered images. These images were presented in rapid succession, creating the appearance of smooth, continuous rotation (Supplementary Movie S1). As for the tactile stimuli, the spiky, neutral, and round shapes were 3D-printed using polyamide material in a white color (EOS, Munich, Germany).

#### Procedures

**Figure 2.**
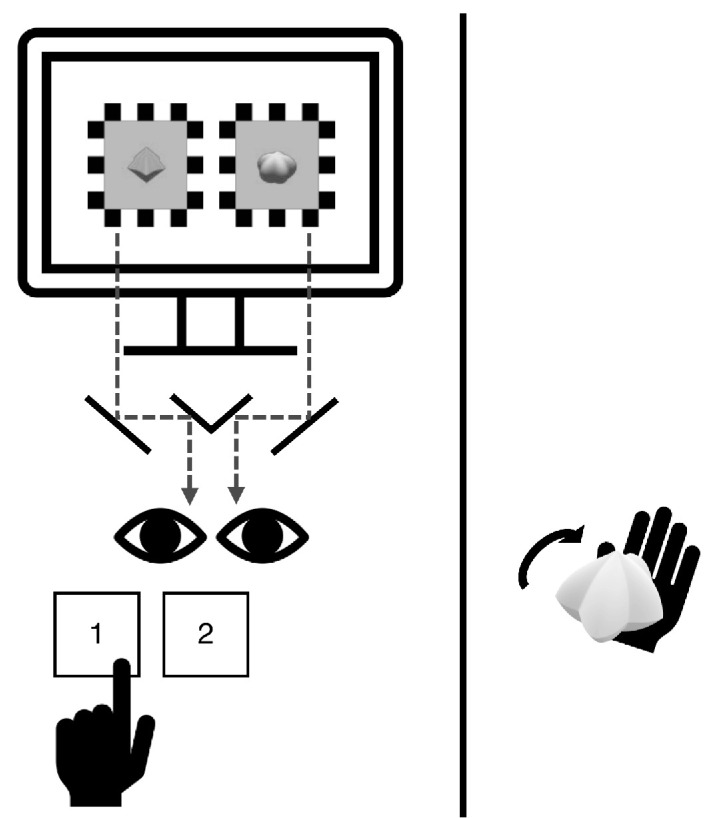
Experiment 1 setting and procedures. Rapid successions of 3D-rendered images resembling continuous rotations of each spiky and round shape were presented to the same retinal positions of the two eyes through a mirror stereoscope. Participants were asked to report their visual experience with the keyboard (1 if spiky dominant, 2 if round dominant) using their left hand. In visuotactile trials, a spiky, neutral, or round tactile stimulus was given to the participants for them to rotate using their right hand. A black curtain was drawn between the participant and the tactile stimulus to prevent viewing it.

The experiment was conducted across 2 days. Upon arrival, participants sat on the left side of the table, and a black curtain was drawn in the middle of the table to ensure that they could not see the tactile stimuli (Figure 2). Calibration procedures were implemented to induce a stable rivalry experience. On the first day of the experiment, a practice session was conducted to familiarize participants with the task and to screen out those showing strong eye dominance.

The main session consisted of three blocks: one visual-only block and two visuotactile blocks. The visual-only block was always conducted first, on the first day of the experiment, followed by one visuotactile block. The remaining visuotactile block was conducted on the second day of the experiment. The two conditions of blocks differed in the sense that the visual-only block presented only visual stimuli during binocular rivalry but the visuotactile blocks simultaneously presented tactile stimulation along with rival visual stimuli.

Regardless of the block condition, the task was the same for every trial: to track the dominant visual percept during binocular rivalry. A simplified version of the calibration could be done before a trial started to ensure the binocular alignment of stimuli throughout the experiment. Pressing the spacebar confirmed the calibration and initiated the trial. When a beep sound signaled the start of rivalry, rapid successions of 3D-rendered images resembling continuous rotations of the spiky stimulus and round stimulus were presented on the monitor. Stimuli were adjusted to be presented onto the same retinal positions of the two eyes and were dichoptically viewed using a mirror stereoscope (Figure 2). A pair of high-contrast checkerboard windows framed the visual targets to promote stable binocular alignment. Participants were instructed to focus on the shape of the visual stimuli and report the current state of visual dominance by pressing the appropriate button with their left hand and holding it down for as long as the percept lasted (1 for spiky, 2 for round). Upon experiencing a mixed percept of two shapes, they were to press both buttons (Figure 2). A trial ended with another beep sound. The duration of a single binocular rivalry trial was approximately 30 seconds, although the trial did not terminate until the participant indicated a switch in percept. The eye location (left/right) for each shape was counterbalanced for each trial. Moreover, the rotations in the two eyes were presented in opposite directions. For instance, if the spiky shape was presented as rotating clockwise, the round shape was presented as rotating counterclockwise.

In the visuotactile blocks, as each trial began, one of the three shape objects was placed in the palm of the the participant’s right hand, which was extended over the curtain. The participant was told to hold and rotate the 3D-printed object with their fingers while engaging in the task (Figure 2). When the trial ended, the stimulus was taken from the participant. In each visuotactile block, the tactile rotation direction was fixed as either clockwise or counterclockwise, differing in the two blocks. Participants were instructed to rotate the tactile stimulus in their hand in the designated direction within a block. This resulted in congruency of the rotation direction of the tactile stimulus with only one of the visual targets. The order of the visuotactile blocks was counterbalanced.

In a total of 160 trials, 40 trials were visual-only trials, and the remaining 120 trials were visuotactile trials. For the visuotactile trials, spiky, neutral, and round tactile shape conditions were presented 40 times each. With regard to rotation direction congruence, in half of the 40 trials for the spiky and round shape conditions the visual target congruent in shape was also congruent in rotation direction. In the other half, the visual target congruent in shape was incongruent in terms of rotation direction. In the 40 neutral shape condition trials, 20 trials consisted of the clockwise rotation of the spiky shape and the counterclockwise rotation of the round shape. In the other 20 trials, this rotation direction was reversed. The order of tactile shape and direction congruence in the visuotactile blocks was randomized. There were 90-second breaks during each block.

#### Analyses

Perceptual experiences of participants during binocular rivalry were analyzed using the indices of predominance, first percept, and normalized dominance duration for each spiky and round visual target. Predominance was defined as the proportion of each visual percept being exclusively dominant along the total duration of a trial. Note that, because a mixed percept of spiky and round could also occur, the predominance values of each exclusive percept do not add up to 100%. To serve the primary aim of the current study to focus on the spiky or round shape congruence of vision and touch, analyses were done focusing on the predominance of each visual percept being exclusively dominant. Analyses regarding the mixed percept proportion were addressed as well, but only briefly and with supplementary purpose. The first percept was the initial percept a participant experiences at the onset of binocular rivalry. The number of times each visual target was first perceived as a dominant percept at trial onset was determined.

For the dominance duration measure, durations either shorter than 300 ms or deviating more than 3 standard deviations from the mean were excluded from the analysis. For normalization purposes in consideration of individual variability of dominance durations (Carter & Pettigrew, 2003; Logothetis, Leopold, & Sheinberg, 1996), the mean dominance duration of each spiky and round shape in the visual-only block was used to normalize the mean dominance durations for the visual percept in the visuotactile blocks. The computed normalized dominance durations were first analyzed into frequency histograms with best-fit gamma distributions. This was done to observe whether the data resembled the characteristic of typical duration distribution reported by previous research (Brascamp, van Ee, Pestman, & van den Berg, 2005).

Predominance was used as a rough measure to observe the overall tendency of dominance, and first percept and normalized dominance duration were the main measures to investigate the detailed dynamics of binocular rivalry regarding visuotactile shape congruence. Therefore, after investigating the general pattern of dominance for each condition using the predominance measure, to serve the main purpose of the current study first percept and normalized dominance duration data were analyzed by pooling the trials into congruent, incongruent, and neutral shape conditions. In general, repeated-measures analyses of variance (ANOVAs) were conducted on each index, with Greenhouse–Geisser correction when Mauchly's test of sphericity indicated that the assumption of sphericity had been violated. Post hoc comparisons using Bonferroni correction were conducted following significant main or interaction effects. In addition, to determine whether the congruence of rotation direction affected rivalry dynamics, analyses including direction congruence were conducted.

### Results and discussion

**Figure 3.**
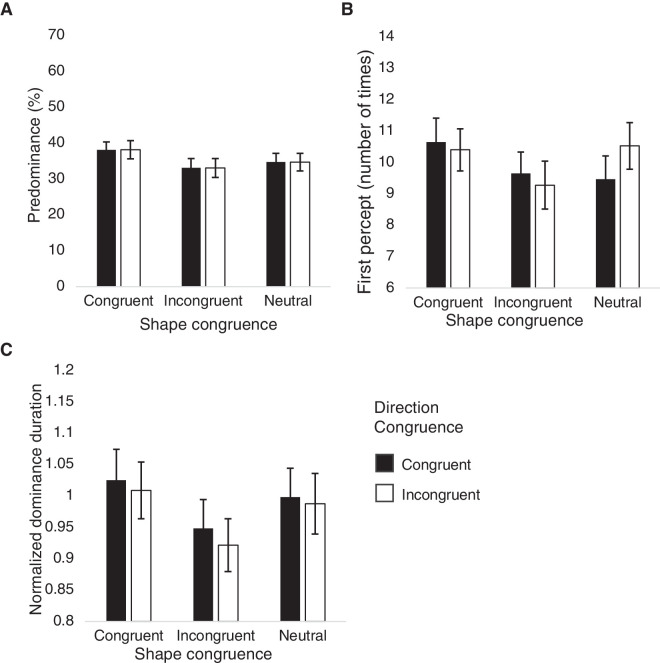
Results including direction congruence from Experiment 1: (**A**) mean predominance values, (**B**) mean number of times of first percepts, and (**C**) mean normalized dominance durations plotted for shape congruence conditions, separately shown by direction congruence.

The effect of rotation direction congruence was examined using two-way repeated-measures ANOVAs with shape congruence and direction congruence as within-participant factors (Figure 3). There were no significant main effects of direction congruence over all indices of predominance, *F*(1, 15) = 0.146, *p* = 0.708, $\eta_{p}^{2}$ = 0.010 (Figure 3A), first percept, *F*(1, 15) = 0.078, *p* = 0.784, $\eta_{p}^{2}$ = 0.005 (Figure 3B), or the normalized dominance duration, *F*(1, 15) = 2.182, *p* = 0.160, $\eta_{p}^{2}$ = 0.127 (Figure 3C). Similarly, results indicated no significant interaction effects of shape congruence and direction congruence for all measures: for predominance, *F*(1.093, 16.398) = 0.534, *p* = 0.491, $\eta_{p}^{2}$ = 0.034; first percept, *F*(1.016, 15.234) = 1.461, *p* = 0.246, $\eta_{p}^{2}$ = 089; or normalized dominance duration, *F*(2, 30) = 0.095, *p* = 0.910, $\eta_{p}^{2}$ = 0.006 (Figure 3). This lack of main and interaction effects regarding direction congruence justified collapsing the data across rotation directions and focusing on shape congruence in the subsequent analyses.

**Figure 4.**
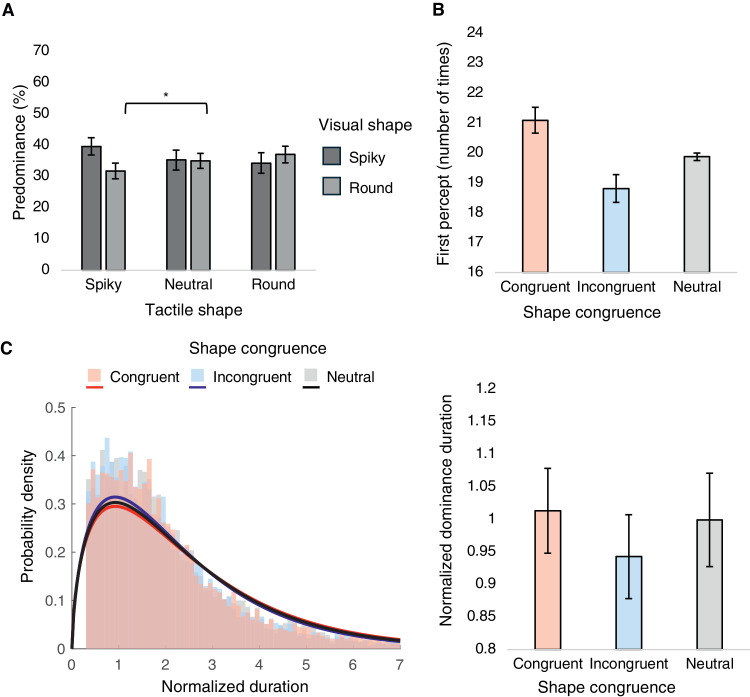
Results from Experiment 1: (**A**) Mean predominance values of spiky and round percepts plotted for tactile shape conditions, with mixed percepts excluded. (**B**) Mean number of times of first percepts plotted for shape congruence conditions. (**C**) Frequency histograms with best-fit gamma distributions of normalized dominance durations (left) and mean normalized dominance durations plotted for shape congruence conditions (right). *p < 0.05.

The spiky percept accounted for 36.39% ± 11.97% and the round percept for 34.60% ± 9.58% of the total perceptual time. A two-way repeated-measures ANOVA on predominance of exclusive percepts was conducted, with visual shape and tactile shape as within-participant factors (Figure 4A). Importantly, a significant interaction effect was revealed, *F*(1.227, 18.401) = 8.245, *p* = 0.007, $\eta_{p}^{2}$ = 0.355. Post hoc analyses indicated a significantly higher predominance of spiky shape when participants touched the spiky shape compared with when they touched the neutral shape (*t* = 3.535, *p* = 0.045, Cohen's *d* = 0.386). Main effects of both visual shape and tactile shape did not reach significance: visual shape, *F*(1, 15) = 0.391, *p* = 0.541, $\eta_{p}^{2}$ = 0.025; tactile shape, *F*(2, 30) = 1.578, *p* = 0.223, $\eta_{p}^{2}$ = 0.095. For completeness, we also examined the proportion of mixed percepts across tactile shape conditions. Although the mixed percepts accounted for 29.00% ± 18.41% of the total perceptual time, the one-way repeated-measures ANOVA revealed no significant effect of tactile shape, *F*(2, 30) = 1.578, *p* = 0.223, $\eta_{p}^{2}$ = 0.095.

A one-way repeated-measures ANOVA with shape congruence as the within-participant factor was conducted on first percept (Figure 4B). The main effect of shape congruence was significant regarding the first percept results, *F*(1.026, 15.391) = 6.352, *p* = 0.023, $\eta_{p}^{2}$ = 0.297; however, the following post hoc comparisons did not reach statistical significance.

Normalized dominance durations for each shape congruence condition all conformed closely to the gamma distribution (Figure 4C, left). Meanwhile, a one-way repeated-measures ANOVA with shape congruence as the within-participant factor did not indicate any significant main effect, *F*(1.349, 20.385) = 2.105, *p* = 0.158, $\eta_{p}^{2}$ = 0.123 (Figure 4C, right). Contrary to our hypothesis, the congruence in shape between visual and tactile stimuli showed limited impact on rivalry dynamics.

To review our experiment setup in line with our hypothesis, we presented successive images of 3D-rendered shapes on the computer screen while the participants actively explored the 3D objects in their hand. One governing principle in successful multisensory integration is the spatial coherence of crossmodal information—crossmodal cues are well integrated when they are perceived to be sharing a common spatial source (Meredith & Stein, 1996; Stein & Stanford, 2008). Considering the spatial disparity between the Experiment 1 visual stimuli (shown on a computer screen) and the tactile stimuli (explored in the participant's outstretched right hand), the insignificant results are not unusual.

Unfortunately, it is impossible to physically match the spatial location of the visual and tactile stimuli under the rivalry paradigm. As an alternative, we decided to replace the visual stimuli to induce a stronger feeling of connection with the tactile stimuli by including bodily cues. Video recordings of a hand touching the 3D-printed tactile object were provided as rivalry stimuli in the following experiment. The appearance of a hand actually touching the given real-world object was expected to play as an additional bodily cue for integration, thus resulting in spatial remapping between vision and touch (Maravita, Spence, & Driver, 2003). It is commonly known from the example of the rubber hand illusion that humans are flexible in their bodily schemas, even in situations where they are perfectly aware that it is a dummy hand (Botvinick & Cohen, 1998; Maravita et al., 2003). Similarly, bodily self-identification could resolve the conflict among vision, touch, and proprioception in the current experimental setup.

Compared with the previous visual stimuli projecting a 3D model rotating midair in virtual space, our new video stimuli depicting a human hand physically touching the 3D-printed stimuli were expected to bias the perception of spatial coherence between the visual and tactile stimuli so that the two senses were colocalized, providing the grounds for successful multisensory integration.

## Experiment 2

The aim of the second experiment was to study the effects of visuotactile integration on binocular rivalry dynamics under active touch conditions in a setup with stronger spatial colocalization between visual and tactile stimuli. Video recordings depicting a hand exploring the 3D-printed spiky and round objects by turning them in the palm with the fingers were used as visual targets. Bodily cues involved in the visual stimuli were thought to lead to illusions in body schemas, thus resulting in colocalization bias (thinking the visual and tactile stimuli were coming from the same location). Simultaneous with the visual stimulation, a spiky, neutral, or round 3D-printed object was presented for each participant to freely explore with their hand. With this change in visual stimulus type, the goal was to study the potentially stronger effects of visuotactile integration on rivalry dynamics. In addition, to focus on the research question of shape congruence, rotation direction was fixed so that it was always congruent between vision and touch, with shape being the sole factor to determine visuotactile congruence. The hypothesis was that the active touch experience of a certain shape would bring the congruent visual percept into relative dominance in binocular rivalry, with a stronger sense of visuotactile spatial coherence rising from the bodily cues in the video-recorded stimuli.

### Methods

#### Participants

Fourteen individuals (nine women; mean age, 29.3 ± 2.6 years; range, 27–33 years) with normal or corrected-to-normal vision participated in the experiment. Because of the design and aim of the experiment to explore the effect of visuotactile integration when touching 3D stimuli, only right-handed participants were recruited. Participants showing strong eye dominance were excluded from the analyses. The study was approved by the Korea University Institutional Review Board (KUIRB-2019-0313-02).

#### Apparatus

The apparatus was identical to that of Experiment 1.

#### Stimuli

Visual stimuli were achromatic video recordings of the experimenter's hand actively touching the 3D-printed objects of each spiky and round shape. A GoPro Hero5 camera (GoPro, Inc., San Mateo, CA) was used to record the videos. The recorded videos displayed the experimenter's right hand holding each stimulus, turning it in the clockwise direction in her palm using her fingers (Supplementary Movie S2). The duration of each video was about 30 seconds. Tactile stimuli were identical to those of Experiment 1.

#### Procedures

**Figure 5.**
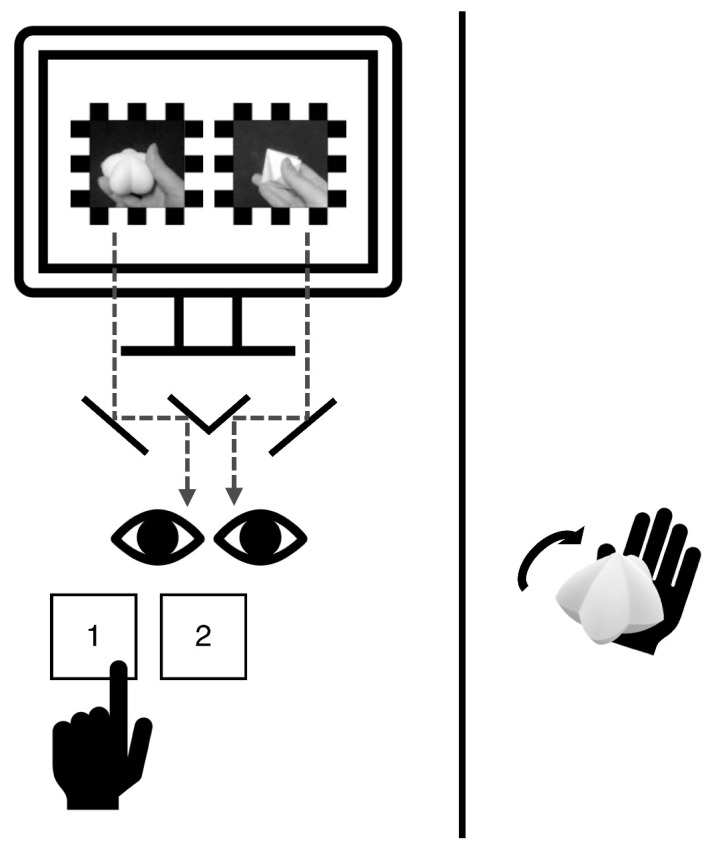
Experiment 2 setting and procedures. Video recordings of a hand rotating each spiky and round shape with the fingers were presented to the same retinal positions of the two eyes through a mirror stereoscope. Participants were asked to report their visual experience with the keyboard (1 if spiky dominant, 2 if round dominant) using their left hand. In visuotactile trials, a spiky, neutral, or round tactile stimulus was given to the participant to rotate using their right hand. A black curtain was drawn between the participant and the tactile stimulus to prevent viewing it.

Compared with Experiment 1, this experiment was shortened and was conducted on a single day. Upon arrival, participants sat on the left side of the table, and a black curtain was drawn in the middle of the table to ensure that they could not see the tactile stimuli (Figure 5). Calibration procedures were implemented to induce a stable rivalry experience. A practice session was conducted to familiarize participants with the task and to screen out those showing strong eye dominance.

The main session consisted of visual-only and visuotactile trials. The two conditions of trials differed in the sense that the visual-only trials presented only visual stimuli during binocular rivalry and that the visuotactile trials simultaneously presented tactile stimulation along with rival visual stimuli. The overall procedure and task for the main session were identical to those of Experiment 1, with the major difference being the visual stimuli. Video recordings of a hand actively touching a spiky tactile stimulus and a round tactile stimulus were dichoptically viewed using a mirror stereoscope (Figure 5). The videos depicted a hand turning the tactile stimulus in the clockwise direction. The duration of a single binocular rivalry trial was approximately 30 seconds, although the trial did not terminate until the participant indicated a switch in percept.

The tactile stimuli in the visuotactile trials were the same as Experiment 1. However, the participant was told to hold and rotate the object in the clockwise direction with their fingers while engaging in the task (Figure 5). Importantly, the rotation direction of the visual and tactile stimuli was fixed and matched as clockwise in every visuotactile trial. In a total of 60 trials, 15 trials were visual-only trials and the remaining 45 trials were visuotactile trials. For the visuotactile trials, spiky, neutral, and round tactile shape conditions were presented 15 times each. The order of the trials was randomized.

#### Analyses

The analysis procedures were identical to those of Experiment 1.

### Results and discussion

**Figure 6.**
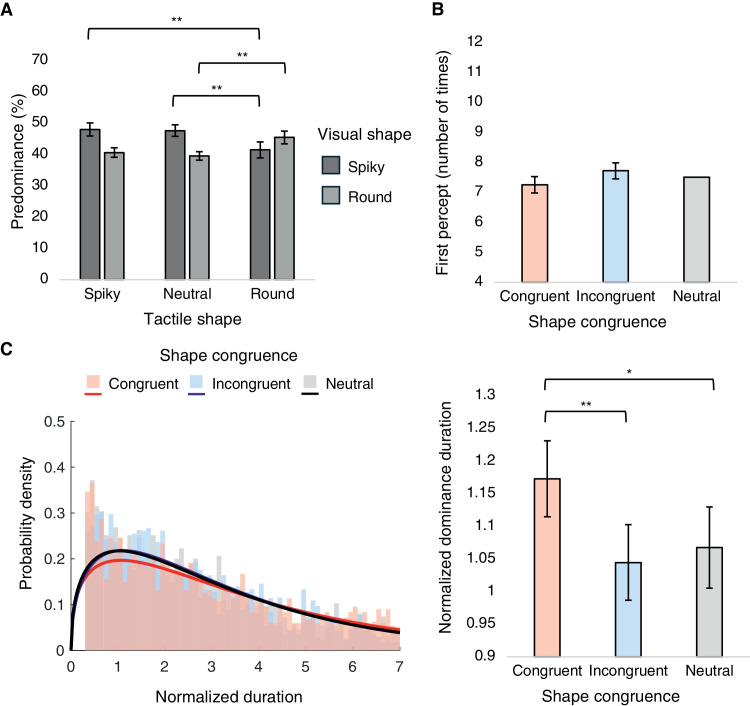
Results from Experiment 2. (**A**) Mean predominance values of spiky and round percepts plotted for tactile shape conditions, with mixed percepts excluded. (**B**) Mean number of times of first percepts plotted for shape congruence conditions. (**C**) Frequency histograms with best-fit gamma distributions of normalized dominance durations (left) and mean normalized dominance durations plotted for shape congruence conditions (right). *p < 0.05, **p < 0.01.

The spiky percept accounted for 45.61% ± 7.67% and the round percept for 41.78% ± 5.44% of the total perceptual time. A two-way repeated-measures ANOVA on predominance of exclusive percepts was conducted, with visual shape and tactile shape as within-participant factors (Figure 6A). Importantly, there was a significant interaction effect, *F*(2, 26) = 13.425, *p* < 0.001, $\eta_{p}^{2}$ = 0.508. Post hoc results indicated significantly higher predominance of spiky shape when touching the spiky shape compared with when touching the round shape (*t* = 4.912, *p* = 0.004, Cohen's *d* = 0.893). In contrast, compared with when touching the neutral shape, the predominance of spiky shape was significantly lower when touching the round shape (*t* = –5.175, *p* = 0.003, Cohen's *d* = –0.843). Finally, the predominance of round shape was higher when touching the round shape compared with when touching the neutral shape (*t* = 4.576, *p* = 0.008, Cohen's *d* = 0.820). The main effects of both visual shape and tactile shape did not reach significance: visual shape, *F*(1, 13) = 1.764, *p* = 0.207, $\eta_{p}^{2}$ = 0.119; tactile shape, *F*(2, 26) = 2.990, *p* = 0.068, $\eta_{p}^{2}$ = 0.187. For completeness, we also examined the proportion of mixed percepts across tactile shape conditions. The mixed percepts accounted for only 12.61% ± 7.79% of the total perceptual time, and the one-way repeated-measures ANOVA revealed no significant effect of tactile shape, *F*(2, 26) = 2.990, *p* = 0.068, $\eta_{p}^{2}$ = 0.187.

A one-way repeated-measures ANOVA with shape congruence as the within-participant factor was conducted on the first percept (Figure 6B); however, the main effect of shape congruence was insignificant, *F*(1.003, 13.038) = 0.751, *p* = 0.402, $\eta_{p}^{2}$ = 0.055.

Normalized dominance durations for each shape congruence condition all conformed closely to the gamma distribution (Figure 6C, left). A one-way repeated-measures ANOVA with shape congruence as the within-participant factor indicated a significant main effect, *F*(2, 26) = 8.431, *p* = 0.002, $\eta_{p}^{2}$ = 0.393 (Figure 6C, right). Post hoc results showed that congruent conditions led to longer dominance durations compared with both incongruent and neutral conditions (congruent−incongruent, *t* = 3.835, *p* = 0.006, Cohen's *d* = 0.576; congruent−neutral, *t* = 3.290, *p* = 0.018, Cohen's *d* = 0.474).

To sum up, our video stimuli that provide cues for colocalization between vision and touch led to significant crossmodal influences during binocular rivalry. This significant crossmodal impact was mainly indicated in predominance and normalized dominance duration indices, illustrating that congruent tactile stimulation in shape can lead to both higher dominance proportions and longer dominance durations compared with situations with incongruent or neutral tactile stimulations. Therefore, the current results provide novel evidence regarding active tactile stimulation and visual disambiguation.

However, in our attempts to address the naturalistic behavior of touch exploration, we instructed participants to freely explore the given object; that is, factors such as specific finger movement patterns or rotation speed were not controlled. Considering the controlled experimental settings in previous investigations, there could be some concern interpreting these results in comparison with them. Therefore, although the voluntary action of perceivers is a meaningful factor in naturalistic 3D object recognition, in the next two experiments we attempted to replicate the research questions under controlled, passive touch conditions with the intent to observe the novel findings of Experiments 1 and 2 within carefully controlled experimental setups more commonly adopted in previous literature.

## Experiment 3

The aim of the third experiment was to study the effects of visuotactile integration on binocular rivalry dynamics under controlled passive touch conditions. The visual stimuli were the same as in Experiment 1—rapid successions of 3D-rendered images of spiky and round shapes. As for the tactile manipulation, participants were to make stationary hand contact with the rotating spiky, neutral, or round 3D-printed objects. Through this passive touch design, additional factors such as the speed, direction, or axis of rotation were matched between the visual and tactile stimuli to eliminate potential confounding factors. The hypothesis was that tactile shape congruence experienced by passive touch would lead to visual dominance in binocular rivalry dynamics. Although Experiment 1 did not yield results supporting this hypothesis, we thought it significant to reinvestigate it under a more controlled, passive touch condition.

### Methods

#### Participants

Twenty-three individuals (12 women; mean age = 24.7 ± 4.8 years; range, 19–38 years) with normal or corrected-to-normal vision participated in the experiment. Because of the design and aim of the experiment to explore the effect of visuotactile integration when touching 3D stimuli, only right-handed participants were recruited. Participants showing strong eye dominance were excluded from the analyses. The study was approved by the Korea University Institutional Review Board (KUIRB-2022-0370-04).

#### Apparatus

The experiment was run in a quiet, dark room. Participants sat in front of a 19-inch CRT monitor (1024 × 768-pixel resolution, 60-Hz refresh rate, 43-cm viewing distance), and the experiment was conducted using MATLAB 2018b and Psychophysics Toolbox version 3 (Brainard, 1997; Pelli, 1997). An Uno R3 board (Arduino, Monza, Italy) was used to control the 6-volt, 16-rpm DC motor (JGA25-370; OEM manufacturing, China) via motor driver (L298N; STMicroelectronics, Plan-les-Ouates, Switzerland). Arduino IDE 1.8.19 was used to program the Arduino board, and stimuli were rotated to provide passive tactile stimulation to the participants.

#### Stimuli

Visual stimuli were the same as those of Experiment 1, which were rapid successions of 3D-rendered images, except for the rotation direction and speed. For both left and right eye stimuli, rotation was always fixed in the clockwise direction. Adjustment in visual rotation speed was done to match that of the tactile stimuli rotation (Supplementary Movie S3). Spiky, round, and neutral tactile stimuli were identical to those of Experiments 1 and 2, except that a pillar and a stand were added to connect the stimuli onto the motor. The three shapes were 3D-printed using polyamide material in a white color (Materialise, Leuven, Belgium). Each stimulus was installed onto the motor to be rotated at a nominal speed of 254 rpm, as specified with the Arduino command. This specific speed value was selected to match the rotation speed of the tactile stimuli with that of the visual stimuli (Supplementary Movie S4). Note that the same spiky, neutral, and round tactile shapes used in Experiments 1 and 2 were tested in advance through a preliminary experiment and were validated to be perceived in the intended curvature within passive touch context.

#### Procedures

The calibration and practice procedures were identical to those of Experiment 2. The main session consisted of four blocks with one visual-only block and three visuotactile blocks. The visual-only block was always conducted first, followed by three visuotactile blocks. The two conditions of blocks differed in the sense that the visual-only block presented only visual stimuli during binocular rivalry and that the visuotactile blocks simultaneously presented tactile stimulation along with rival visual stimuli.

**Figure 7.**
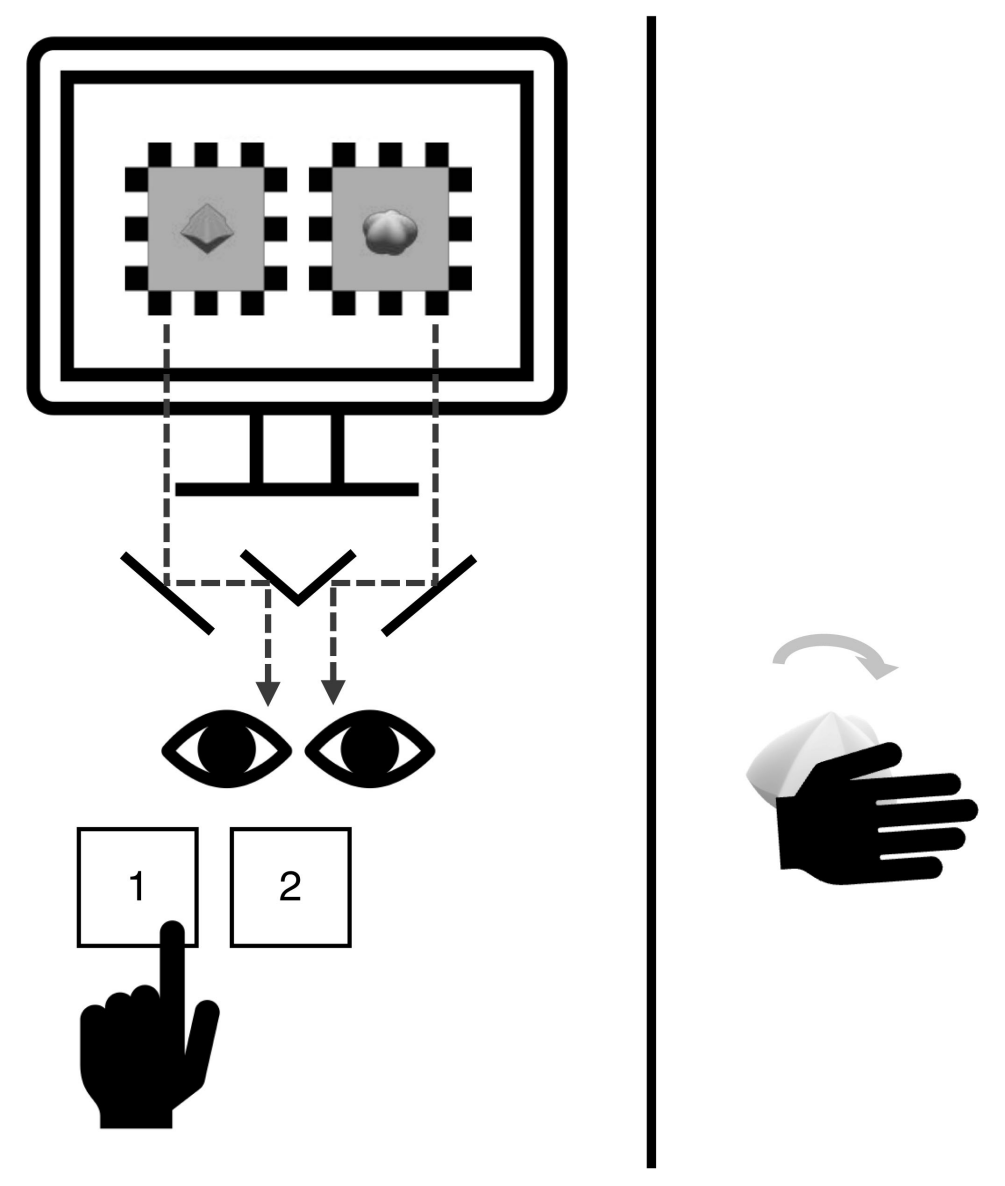
Experiment 3 setting and procedures. Rapid successions of 3D-rendered images that resemble continuous rotations of each spiky and round shape were presented to the same retinal positions of the two eyes through a mirror stereoscope. Participants were asked to report their visual experience with the keyboard (1 if spiky dominant, 2 if round dominant) using their left hand. In visuotactile trials, a spiky, neutral, or round tactile stimulus was installed on the motor and rotated, and the participants were asked to make stationary hand contact with it using their right hand. Importantly, the rotation direction and speed matched that of the visual stimuli. A black curtain was drawn between the participant and the tactile stimulus to prevent viewing it.

Regardless of the block condition, the task was the same for every trial—to track the dominant visual percept during binocular rivalry. The specifics were mostly identical to those of Experiment 1 (Figure 7), but the rotation direction of the visual stimuli was fixed in the clockwise direction. The duration of a single trial lasted for approximately 38 seconds.

In the visuotactile blocks, as each trial began, one of the three tactile stimuli was installed on the motor and rotated, and the participant was told to reach out their right arm over the curtain to enclose the tactile stimulus in their right hand. Participants were instructed to minimize hand movements and to feel the tactile stimulation given from the shape of the rotating object while engaging in the task (Figure 7). When the trial ended, the rotation stopped, and the participant was instructed to retrieve their hand.

In a total of 80 trials, 20 trials were visual-only trials, and the remaining 60 trials were visuotactile trials. For the visuotactile trials, spiky, neutral, and round tactile shape conditions were presented 20 times each. The order of the trials regarding the tactile shape condition in the visuotactile blocks was randomized. There were 90-second breaks between blocks.

#### Analyses

Predominance and first percept indices were computed with procedures identical to those of Experiments 1 and 2. As for the dominance duration measure, dominance durations that were ongoing at the end of each trial were considered truncated durations and were additionally excluded. Other analysis procedures were identical to those of Experiments 1 and 2. The general method for statistical tests was identical to those of Experiments 1 and 2.

### Results and discussion

**Figure 8.**
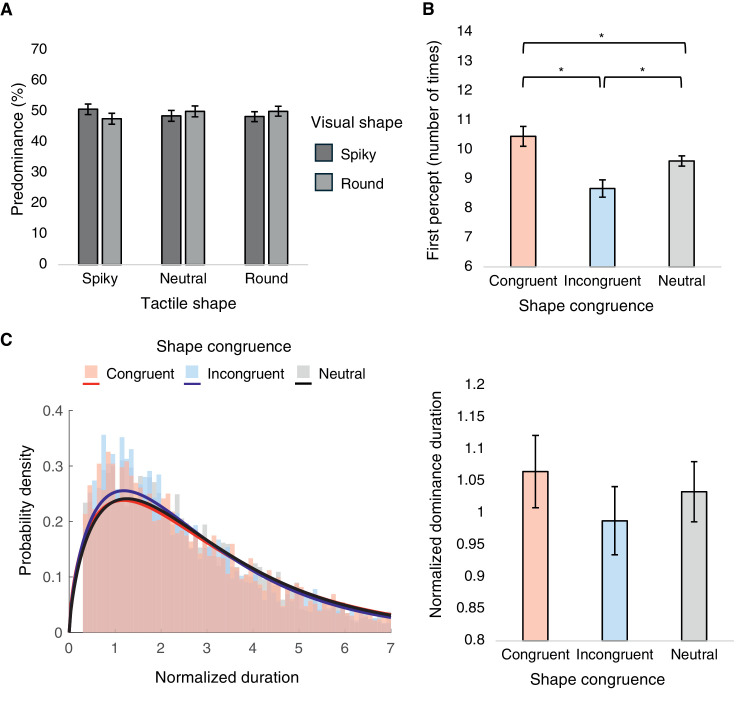
Results from Experiment 3. (**A**) Mean predominance values of spiky and round percepts plotted for tactile shape conditions, with mixed percepts excluded. (**B**) Mean number of times of first percepts plotted for shape congruence conditions. (**C**) Frequency histograms with best-fit gamma distributions of normalized dominance durations (left) and mean normalized dominance durations plotted for shape congruence conditions (right). *p < 0.05.

The spiky percept accounted for 49.15% ± 7.44% and the round percept for 49.21% ± 7.51% of the total perceptual time. A two-way repeated-measures ANOVA on predominance of exclusive percepts was conducted, with visual shape and tactile shape as within-participant factors (Figure 8A). However, the interaction effect was not significant, *F*(2, 44) = 2.725, *p* = 0.077, $\eta_{p}^{2}$ = 0.110, nor were the main effects: visual shape, *F*(1, 22) = 3.495 × 10^–^^4^, *p* = 0.985, $\eta_{p}^{2}$ = 1.589 × 10^–^^5^; tactile shape, *F*(1.173, 25.807) = 0.204, *p* = 0.694, $\eta_{p}^{2}$ = 0.009. For completeness, we also examined the proportion of mixed percepts across tactile shape conditions. The mixed percepts accounted for only 1.64% ± 3.17% of the total perceptual time, and the one-way repeated-measures ANOVA revealed no significant effect of tactile shape, *F*(1.173, 25.807) = 0.204, *p* = 0.694, $\eta_{p}^{2}$ = 0.009.

A one-way repeated-measures ANOVA with shape congruence as the within-participant factor was conducted on first percept (Figure 8B). The main effect of shape congruence was significant, *F*(1.063, 23.391) = 9.664, *p* = 0.004, $\eta_{p}^{2}$ = 0.305. Post hoc analyses revealed significant differences among the congruent and incongruent conditions (*t* = 3.156, *p* = 0.014, Cohen's *d* = 1.332), the congruent and neutral conditions (*t* = 2.852, *p* = 0.028, Cohen's *d* = 0.633), and, finally, the incongruent and neutral conditions (*t* = –3.183, *p* = 0.013, Cohen's *d* = –0.698).

Normalized dominance durations for each shape congruence condition all conformed closely to the gamma distribution (Figure 8C, left). Meanwhile, a one-way repeated-measures ANOVA with shape congruence as the within-participant factor did not indicate any significant main effect, *F*(1.349, 20.385) = 2.105, *p* = 0.158, $\eta_{p}^{2}$ = 0.123 (Figure 8C, right).

To sum up, only the first percept index showed results in line with our hypothesis. Predominance and normalized dominance duration measures did not suggest the influence of visuotactile shape congruence in rivalry dynamics. This weak evidence was in line with the insignificant results of Experiment 1. The visual targets of 3D models rotating midair are thought to have weak relevance to the 3D objects participants are exploring in their hands, without bodily cues to hint at colocalization between visual and touch experiences. Therefore, in our last experiment, we used video recordings as visual targets to encourage spatial colocalization between the two modalities and boost visuotactile integration, under passive touch contexts.

## Experiment 4

The aim of the last experiment was to study the effects of visuotactile integration on binocular rivalry dynamics under controlled passive touch conditions using video recordings as visual targets. The new visual stimuli were similar to the stimuli used in Experiment 2, except that they displayed passive touch. Video recordings showed a stationary human hand making contact with rotating 3D-printed spiky and round objects. The bodily cues embedded in the visual targets were expected to heighten the perception of colocalization between the visual and tactile stimuli, therefore promoting visuotactile integration. For the tactile manipulation, participants were instructed to keep their hand stationary and steady as the rotating spiky, neutral, or round 3D-printed objects came into contact, in the same way as the video recordings. The hypothesis was that passively touching a certain tactile shape would bring the congruent video target into relative dominance in binocular rivalry to a stronger degree, with the experimental setting enhancing visuotactile colocalization compared with the previous experiment. The aim of this passive touch design was to eliminate potential confounds from the active touch conditions in Experiments 1 and 2. This could also provide more comprehensive insights when interpreted together with previous literature.

### Methods

#### Participants

Twenty-four individuals (15 women; mean age = 22.8 ± 2.7 years; range, 19–30 years) with normal or corrected-to-normal vision participated in the experiment. Because of the design and aim of the experiment was to explore the effect of visuotactile integration when touching 3D stimuli, only right-handed participants were recruited. Participants showing strong eye dominance were excluded from the analyses. The study was approved by the Korea University Institutional Review Board (KUIRB-2022-0370-04).

#### Apparatus

The apparatus was identical to that of Experiment 3.

#### Stimuli

Visual stimuli were achromatic video recordings of the experimenter making stationary hand contact with rotating 3D-printed objects of each spiky and round shape. A GoPro Hero5 camera was used to record the videos. Using the same experimental setup as that of Experiment 3, the recorded videos displayed each stimulus installed on the motor and rotating clockwise, with the experimenter's hand enclosed around it (Supplementary Movie S5). While minimizing hand movements, the experimenter's hand maintained contact with the rotating stimulus throughout the entire duration of the video, which lasted about 38 seconds. Tactile stimuli were identical to those of Experiment 3.

#### Procedures

**Figure 9.**
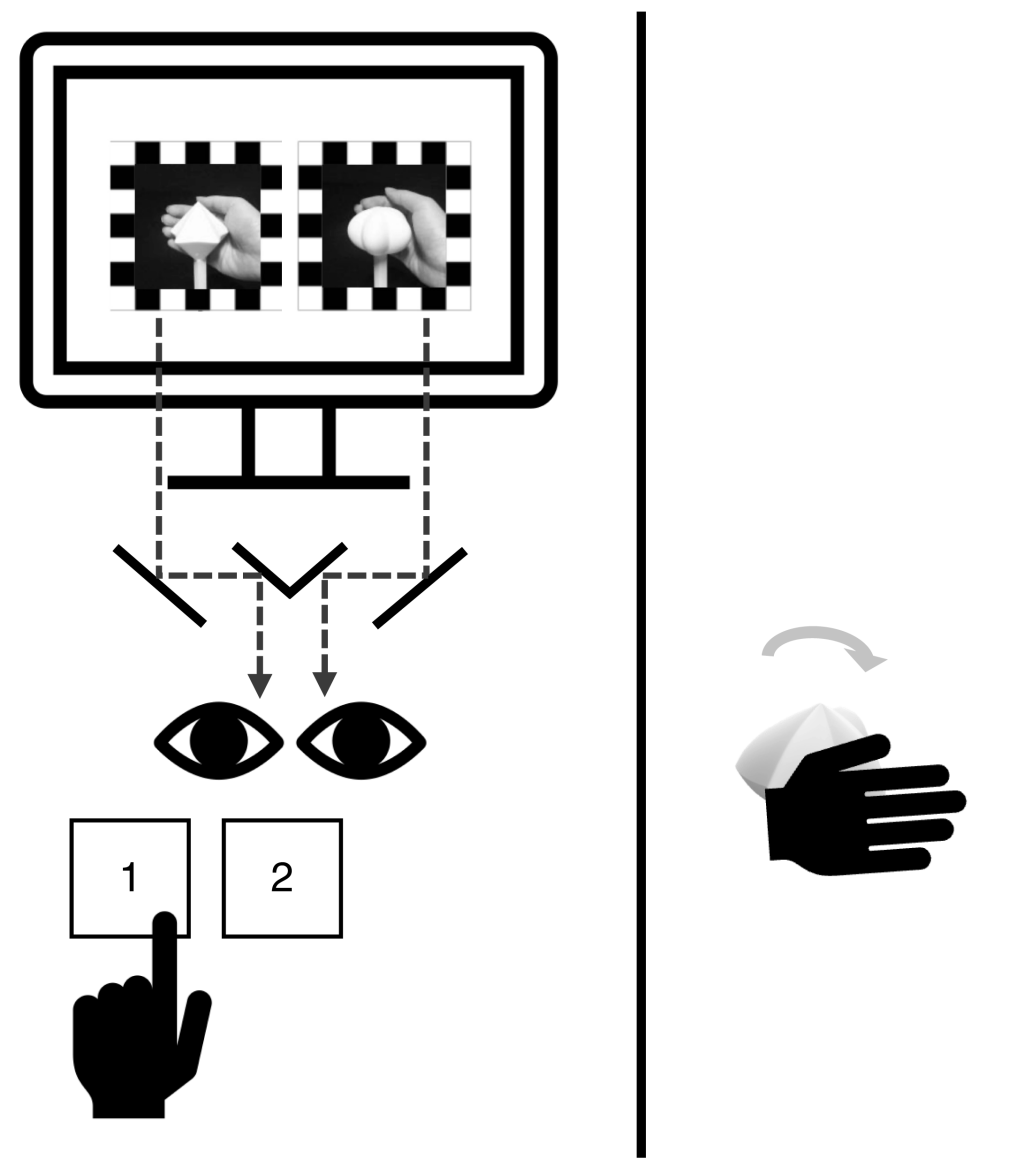
Experiment 4 setting and procedures. Video recordings of a hand making stationary contact with each rotating spiky and round shape were presented to the same retinal positions of the two eyes through a mirror stereoscope. Participants were asked to report their visual experience with the keyboard (1 if spiky dominant, 2 if round dominant) using their left hand. In visuotactile trials, a spiky, neutral, or round tactile stimulus was installed on the motor and rotated, and the participants were asked to make stationary hand contact with it using their right hand. A black curtain was drawn between the participant and the tactile stimulus to prevent viewing it.

The overall procedures were identical to those of Experiment 3, with the major difference being the visual stimuli. Video recordings of a hand making stationary contact with a rotating spiky tactile stimulus and with a rotating round tactile stimulus were dichoptically viewed using a mirror stereoscope (Figure 9).

#### Analyses

The analysis procedures were identical to those of Experiment 3.

### Results and discussion

**Figure 10.**
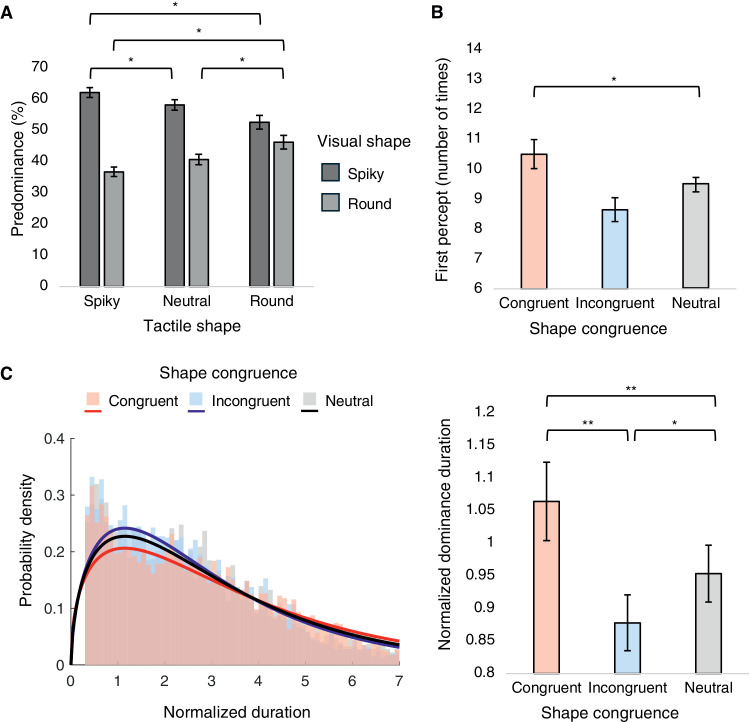
Results from Experiment 4. (**A**) Mean predominance values of spiky and round percepts plotted for tactile shape conditions, with mixed percepts excluded. (**B**) Mean number of times of first percepts plotted for shape congruence conditions. (**C**) Frequency histograms with best-fit gamma distributions of normalized dominance durations (left) and mean normalized dominance durations plotted for shape congruence conditions (right). *p < 0.05, **p < 0.01.

The spiky percept accounted for 57.50% ± 7.50% and the round percept for 41.13% ± 7.42% of the total perceptual time. A two-way repeated-measures ANOVA on predominance of exclusive percepts was conducted, with visual shape and tactile shape as within-participant factors (Figure 10A). Importantly, there was a significant interaction effect, *F*(1.136, 26.122) = 15.710, *p* < 0.001, $\eta_{p}^{2}$ = 0.406. However, the main effect of visual shape was also highly significant, *F*(1, 23) = 29.041, *p* < 0.001, $\eta_{p}^{2}$ = 0.558, with post hoc tests indicating a trend of spiky percept predominating over the round percept (*t* = 5.389, *p* < 0.001, Cohen's *d* = 1.846). Thus, only the significant comparisons indicating the effect of shape congruence between vision and touch, rather than the strong dominance of spiky percept itself, were focused. The predominance of spiky percept was higher when touching the spiky shape compared with when touching the round shape (*t* = 4.063, *p* = 0.007, Cohen's *d* = 1.066) or neutral shape (*t* = 3.306, *p* = 0.046, Cohen's *d* = 0.443). Similarly, the predominance of round percepts was higher when touching the round shape compared with when touching the spiky shape (*t* = 4.100, *p* = 0.007, Cohen's *d* = 1.068) or neutral shape (*t* = 4.086, *p* = 0.007, Cohen's *d* = 0.621). The main effect of tactile shape was insignificant, *F*(2, 46) = 0.108, *p* = 0.898, $\eta_{p}^{2}$ = 0.005. For completeness, we also examined the proportion of mixed percepts across tactile shape conditions. The mixed percepts accounted for only 1.37% ± 1.00% of the total perceptual time, and the one-way repeated-measures ANOVA revealed no significant effect of tactile shape, *F*(2, 46) = 0.108, *p* = 0.898, $\eta_{p}^{2}$ = 0.005.

A one-way repeated-measures ANOVA with shape congruence as the within-participant factor was conducted on the first percept (Figure 10B). The main effect of shape congruence was significant, *F*(1.032, 23.726) = 5.873, *p* = 0.023, $\eta_{p}^{2}$ = 0.203. Post hoc results indicated a significant difference between the congruent and neutral conditions (*t* = 2.793, *p* = 0.031, Cohen's *d* = 0.538).

Normalized dominance durations for each shape congruence condition all conformed closely to the gamma distribution (Figure 10C, left). A one-way repeated-measures ANOVA with shape congruence as the within-participant factor indicated a significant main effect, *F*(1.232, 28.332 = 14.927, *p* < 0.001, $\eta_{p}^{2}$ = 0.394 (Figure 10C, right). Post hoc analyses revealed significant differences among the congruent and incongruent conditions (*t* = 4.067, *p* = 0.001, Cohen's *d* = 0.769), the congruent and neutral conditions (*t* = 3.892, *p* = 0.002, Cohen's *d* = 0.457), and, finally, the incongruent and neutral conditions (*t* = –3.034, *p* = 0.018, Cohen's *d* = –0.312).

The results indicated strong crossmodal effects of shape congruence on visual disambiguation, consistently reported over all indices of predominance, first percept, and normalized dominance duration. In line with the hypothesis, touching a 3D-printed object led to relative dominance of the visual percept that is congruent in shape.

By introducing video recordings with bodily cues so participants would perceive the presented hand as theirs, we enhanced spatial colocalization of vision and touch. The results provided clear insights into the crossmodal disambiguation process between vision and touch in 3D object recognition. This evidence is consistent with many previous studies proposing the beneficial effects of visuotactile congruence on perceptual disambiguation.

## Discussion

**Figure 11.**
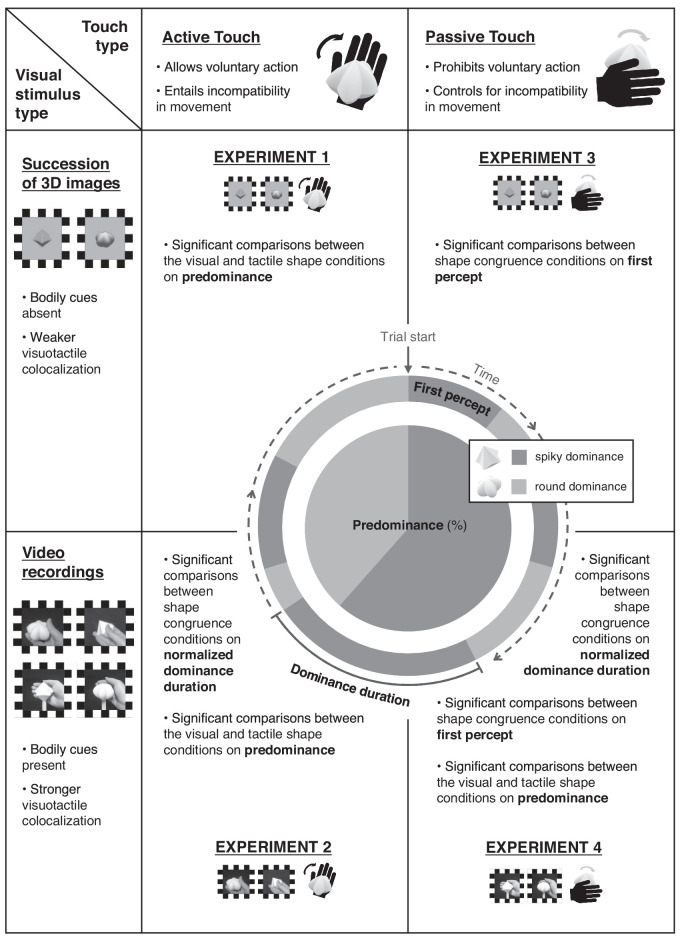
Summary of the main findings in Experiments 1 to 4. Experiments could be divided in terms of touch type and visual stimulus type. Experiments 1 and 2 employed active touch, and Experiments 3 and 4 employed passive touch. Experiments 1 and 3 used rapid successions of 3D images for visual stimuli, and Experiments 2 and 4 used video recordings for visual stimuli. The characteristics of each design manipulation are described. Using the binocular rivalry paradigm, the indices including predominance, first percept, and normalized dominance duration were observed regarding the spiky and round shape dominance. The diagram in the middle visualizes the definition and computation of each measure by illustrating an example trial of rivalry experience. The results of each experiment are summarized in each box.

The present study aimed to investigate the visuotactile crossmodal congruence effects on binocular rivalry dynamics. To test for shape congruence in curvature, spiky, neutral, and round shapes were used as 3D tactile stimuli to aid in disambiguation of dichoptically presented spiky and round visual stimuli. The overall results across the four experiments are summarized in Figure 11. Experiment 1 encouraged the voluntary actions of participants to explore the tactile object freely with their hands and fingers but suggested weak evidence regarding shape congruence. We suspected that the results could be due to the spatial disparity between visual and tactile stimuli that inevitably arises from the rivalry paradigm. Thus, in Experiment 2, visual stimuli were replaced by video recordings that displayed a hand touching the tactile object to work as a bodily cue to bias the impression of crossmodal spatial coherence. The results showed stronger dominance for the visual target with active exploration of a congruent tactile object. Meanwhile, our experimental setting of encouraging free exploratory action inevitably led to incompatibility in movement factors between vision and touch. Such inconsistency between the visual and tactile experiences could lead to confounding factors and diverge from the controlled setup of existing literature. Therefore, in Experiments 3 and 4, we attempted to replicate the findings under passive touch conditions, which were experienced by making stationary hand contact with a rotating tactile object. Whereas Experiment 3 using 3D images as visual stimuli yielded significant results only for the first percept measure, the video-recorded stimuli in Experiment 4 led to prominent shape congruence effects across all indices of predominance, first percept, and normalized dominance duration. Overall, from experimental settings that encouraged spatial colocalization and thus successful multisensory integration, the results suggest that simultaneous tactile experience of both active and passive touch can lead to dominance of the relevant visual target during binocular rivalry.

The results are in line with previous studies that have investigated visuotactile congruence effects during binocular rivalry, in the sense that rival targets with crossmodally congruent features are boosted to become dominant (Hense et al., 2019; Liaw et al., 2022; Lunghi & Alais, 2013; Lunghi & Alais, 2015; Lunghi & Morrone, 2013; Lunghi et al., 2010; Lunghi et al., 2014; van Ee et al., 2009). For example, van Ee et al. (2009) extended the crossmodally congruent benefits from the audiovisual to the visuotactile domain along a series of experiments, demonstrated by longer dominance for targets with the same movement pattern as the sound and tactile vibration. In the current study, we did not analyze suppression duration separately because, in our continuous-report paradigm, dominance and suppression simply reflected opposite sides of the same congruence manipulation. However, several studies have also reported the existence of crossmodal interaction during the suppression state (Lunghi & Alais, 2015; Lunghi et al., 2010). Overall, our results add support for a beneficial influence of visuotactile integration on disambiguation during binocular rivalry, suggesting that shape information in the tactile modality can increase the dominance of the congruent visual target.

In addition, the current study examined the novel 3D nature of stimuli. In previous literature, orientation has most often been manipulated across visuotactile modalities (Lunghi & Alais, 2013; Lunghi & Alais, 2015; Lunghi & Morrone, 2013; Lunghi et al., 2010; Lunghi et al., 2014). Other works have focused on motion direction or motion pattern (Hense et al., 2019; Liaw et al., 2022; van Ee et al., 2009). Compared with such 1D or 2D stimuli, the 3D shapes used in this study closely resembled objects in our daily lives. A recent review pointed out that 3D stimuli are different from 2D stimuli in the nature of their behavioral and neural processing (Kyler, 2024). It has been suggested that 3D stimuli are better perceived, recognized, or remembered compared with 2D stimuli (Korisky & Mudrik, 2021; Shimizu, Saida & Shimura, 1993; Snow, Gomez, & Compton, 2023; Snow, Skiba, Coleman, & Berryhill, 2014). Some studies have also reported that the two stimuli types differ in their neural mechanisms of repetition adaptation (Snow et al., 2011). In the current study, rather than conventional 1D or 2D stimuli, we chose 3D objects to add valuable object-level insights on visuotactile integration.

Moreover, the present study gains novelty in terms of including voluntary action in investigating visuotactile interaction. Although tactile experiences can be passive, active touch with exploratory muscle movements can better represent naturalistic haptic experiences. Still, not many studies have explored how exploratory muscle movements can influence visuotactile perceptual disambiguation. A study by Bruno et al. (2007) proposed that active touch led to longer durations and fewer reversals when perceiving a Necker cube compared with passive touch. Although the researchers managed to build a 3D Necker cube with wires, the study still carries limitations in the sense that their results are only interpretable within the particular bistable phenomenon of the Necker cube. Meanwhile, Lunghi and Morrone (2013) dissociated between the active exploration and passive touch conditions during binocular rivalry, and their results suggest the equally effective influence of tactile stimulation in perceptual disambiguation. However, their active exploration condition was defined as stroking the sinusoidal grating stimuli with the right index finger, which differs in the nature of the stimulus from the 3D stimuli used in the current study. In contrast, in the active touch experiments of the current study participants held the tactile objects in the palm of the hand and used all five fingers to grasp and rotate the object in a fluid motion (without the help of any device or external influence). These movements are analogous to how we would explore a real-life object in a naturalistic environment and thus carries advantage when investigating perception as an active and interactive process (Hommel, 2009; Schroeder, Wilson, Radman, Scharfman, & Lakatos, 2010).

Despite this significance, active touch did not yield a clearly distinguishable impact over passive touch; the results were overall comparable between the two modes of tactile exploration. It is difficult to draw a firm conclusion, however, because additional confounding factors prevented direct statistical comparisons between active and passive touch. That is, due to the unrestricted nature of voluntary action, a certain degree of incompatibility occurred between vision and touch in the active touch conditions of Experiments 1 and 2. Specifically, factors such as rotation speed, direction, or muscle movement may have acted as potential confounding factors compared with the controlled experimental settings of previous studies. Indeed, a study suggested that the tight temporal coupling of action and its consequences, which they referred to as “contingency,” was critical for perceptual benefits in visuotactile integration (Suzuki, Schwartzman, Augusto, & Seth, 2019). Because visuotactile contingency is inherently intertwined with voluntary action, we could not directly isolate the pure influence of active compared with passive touch in the present design. Similar to the virtual reality setups that reflected participant behavior in real time (Suzuki et al., 2019), building an interactive experimental environment that ensures the tight coupling between visuotactile experiences would be beneficial in future research to enable direct comparisons between the two modes.

In the current study, spatial colocalization is another factor that could explain the results. The spatial rule is a core principle in multisensory integration—that there is a higher possibility for multisensory integration when the stimuli are perceived to be coming from the same spatial source (Meredith & Stein, 1996; Stein & Stanford, 2008). By introducing the video of a human hand touching 3D objects as the visual target, we added the bodily cues of a hand exploring the object. In previous research of the rubber hand illusion, people acted as though the presented rubber hand was their own, demonstrating the malleable nature of proprioception (Botvinick & Cohen, 1998; Lloyd, 2007; Maravita et al., 2003). In the same vein, the added context of a hand exploring objects in Experiments 2 and 4 provided the necessary cues for participants to perceive the visual and tactile information as colocalized. Under this environment of visuotactile spatial coherence, the current study suggests benefits of multisensory integration in perceptual disambiguation, in both active and passive exploration contexts.

The current findings not only contribute to our understanding of behavioral dynamics in visual disambiguation but also offer important implications for the neural mechanisms of binocular rivalry and visuotactile integration. Binocular rivalry has long been considered a useful tool to study the neural correlates of visual perception and awareness (Blake, 1989; Blake & Logothetis, 2002; Tong, Meng, & Blake, 2006). Competition between the two rival eyes are thought to be primarily resolved in the early visual areas such as V1 (Blake, 1989); however, it is also suggested that input signals from other brain regions can influence these early neural representations (Tong et al., 2006). Indeed, relevant studies have suggested that the link between the somatosensory and visual systems is the neural mechanism underlying the intervention of touch in binocular rivalry (Lunghi & Alais, 2013; Lunghi & Alais, 2015; Lunghi et al., 2010; Lunghi et al., 2014). As for the current study, the lateral occipital complex (LOC) can additionally be discussed as the candidate region to produce feedback inputs to resolve the competitive neural signals. Although initially considered a visual region, the LOC is now reported to represent shape information in both visual and tactile modalities and is highlighted for its processing in object-level stimuli (Amedi, Malach, Hendler, Peled, & Zohary, 2001; James, Humphrey, Gati, Menon, & Goodale, 2002; Lacey & Sathian, 2014; Pietrini et al., 2004; Ptito et al., 2012; Reed, Shoham, & Halgren, 2004). Thus, considering the 3D stimuli characteristics in the current study, the top–down feedback signals from the LOC region could be a possible disambiguating factor that influenced the neural representations in the early visual areas.

Taken together, the present study expands the understanding regarding visuotactile integration in perceptual disambiguation, especially in terms of 3D object processing. By examining active versus passive touch and comparing 3D image successions and video recordings, the roles of voluntary action and spatial colocalization on successful integration were investigated. When spatial coherence between vision and touch was encouraged from bodily cues displayed in the video recording stimuli, concurrent tactile experience could boost the congruent visual shape into dominance in binocular rivalry, compared with the incongruent rival shape. In such situations of visuotactile colocalization, both active and passive touch led to beneficial influences in disambiguation. The results provide a basis for further advancements in investigating the naturalistic behavior of humans by utilizing 3D objects as stimuli and encouraging voluntary actions of the perceivers.

## Supplementary Material

Supplement 1

Supplement 2

Supplement 3

Supplement 4

Supplement 5
